# *N*,*N*′-alkylated Imidazolium-Derivatives Act as Quorum-Sensing Inhibitors Targeting the *Pectobacterium atrosepticum*-Induced Symptoms on Potato Tubers

**DOI:** 10.3390/ijms141019976

**Published:** 2013-10-08

**Authors:** Yannick Raoul des Essarts, Mohamad Sabbah, Arnaud Comte, Laurent Soulère, Yves Queneau, Yves Dessaux, Valérie Hélias, Denis Faure

**Affiliations:** 1 Centre National de la Recherche Scientifique, Institut des Sciences du Végétal, UPR 2355, Gif-sur-Yvette 91198, France; E-Mails: yannick.desessarts@isv.cnrs-gif.fr (Y.R.E.); Yves.Dessaux@isv.cnrs-gif.fr (Y.D.); 2 FN3PT/RD3PT, Fédération Nationale des Producteurs de Plants de Pomme de terre, 43-45 Rue de Naples, Paris F-75008, France; E-Mail: valerie.helias@fnpppt.fr; 3 INSA Lyon, ICBMS, UMR 5246, CNRS, Université Lyon 1, INSA-Lyon, CPE-Lyon, Bât J. Verne, 20 av A. Einstein, 69621 Villeurbanne Cedex, France; E-Mails: mohamadsabbah7@hotmail.com (M.S.); laurent.soulere@insa-lyon.fr (L.S.); yves.queneau@insa-lyon.fr (Y.Q.); 4 Service de Chimiothèque, ICBMS, UMR 5246, CNRS, Université Lyon 1, INSA-Lyon, CPE-Lyon, Bât Curien, 43 bd du 11 Novembre 1918, 69622 Villeurbanne Cedex, France; E-Mail: arnaud.comte@univ-lyon1.fr; 5 Institut National de la Recherche Agronomique, UMR 1349IGEPP, Le Rheu F-35653, France

**Keywords:** *N*-acylhomoserine lactone, quorum-sensing, quorum-sensing inhibitor, *Pectobacterium*, soft-rot, potato tuber

## Abstract

Bacteria belonging to the *Pectobacterium* genus are the causative agents of the blackleg and soft-rot diseases that affect potato plants and tubers worldwide. In *Pectobacterium*, the expression of the virulence genes is controlled by quorum-sensing (QS) and *N*-acylhomoserine lactones (AHLs). In this work, we screened a chemical library of QS-inhibitors (QSIs) and AHL-analogs to find novel QSIs targeting the virulence of *Pectobacterium*. Four *N*,*N*′-bisalkylated imidazolium salts were identified as QSIs; they were active at the μM range. In potato tuber assays, two of them were able to decrease the severity of the symptoms provoked by *P. atrosepticum*. This work extends the range of the QSIs acting on the *Pectobacterium*-induced soft-rot disease.

## 1. Introduction

Causative agents of the blackleg and soft-rot diseases of potato belong to the *Pectobacterium* and *Dickeya* genera [1]. These soft-rot enterobacteria produce *N*-acyl homoserine lactones (AHLs), mainly 3-oxo-octanoyl-l-homoserine lactone (3-OC8-HSL) [2,3]. In *P. carotovorum* subsp. *carotovorum* and *P. atrosepticum* populations, AHLs are involved in the expression of virulence factors, including plant cell-wall degrading enzymes, such as cellulases and pectinases [4,5]. This cell-to-cell communication that involves the production, exchange and perception of AHL signals is termed quorum sensing (QS) [6].

Several quorum-quenching strategies have been proposed to interfere with the QS-regulated expression of the virulence factors in *Pectobacterium*. They encompass the construction of transgenic plants that express bacterial AHL-degrading enzymes, such as lactonases [7], the identification and biostimulation of soil AHL-degrading bacteria that could act as biocontrol agents, such as *Bacillus thuringiensis* and *Rhodococcus erythropolis* [8–10], and the identification and synthesis of natural and synthetic compounds acting as quorum-sensing inhibitors (QSIs) [11–13]. In contrast with the abundant literature on QSIs targeting the human pathogen *Pseudomonas aeruginosa* [14–16], only a few QSIs that efficiently reduce the *Pectobacterium*-induced symptoms have been described. Noticeably, some archetypical QSIs active on *Pseudomonas* or other pathogens do not diminish the severity of the *Pectobacterium*-induced symptoms [17], a feature that stresses the importance of the identification of dedicated QSIs targeting this plant pathogen.

In this work, we constructed and used a *Pectobacterium* AHL-biosensor to screen a collection of synthetic AHL and QSI derivatives and identifying QSIs of which the protective activity against the *Pectobacterium-*induced symptoms was evaluated in potato-tuber maceration assays.

## 2. Results and Discussion

### 2.1. Construction of the Pectobacterium AHL-Biosensor

We constructed a *Pectobacterium* AHL-biosensor that exhibited the two typical characteristics of the current QS signals biosensor, *i.e.*, (i) it was defective for the synthesis of its own AHL signal; (ii) it was able to produce a measurable reporting activity that correlated with the concentrations of the added AHLs in the culture medium. In *P. atrosepticum* CFBP6276, the genome sequence of which has been published [18], the *expI* gene encodes the synthase responsible for the biosynthesis of the AHL-signals that are required for the expression of the virulence factors and induction of the plant symptoms on potato tubers [5]. In the *expI* mutant CFBP6276-EI [19], we introduced the plasmid pME6031-*rsmA::uidA* that was generated by cloning the *rsmA::uidA* reporting fusion in the broad range vector pME6031. In *P. atrosepticum*, the *rsmA*-promoter is down-regulated in the presence of AHLs [20]. Hence, in the resulting *Pectobacterium* AHL-biosensor, the *uidA*-encoded glucuronidase activity was expressed at a high level in the absence of AHLs, and decreased after addition of AHLs in the culture medium. QSI molecules should therefore increase the expression of glucuronidase in the presence of AHLs.

### 2.2. QSIs Identification

**Figure 1:**
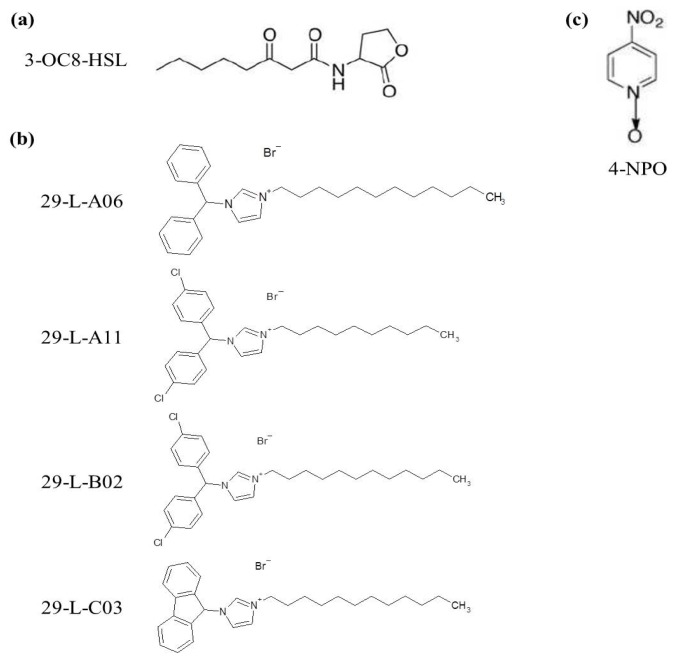
Structure of the used quorum sensing (QS)-molecule and identified quorum sensing-inhibitors (QSIs). (**a**) The 3-OC8-HSL is the *N*-acyl homoserine lactone (AHL) used as the QS-signal. (**b**) Structure of the identified QSIs. (**c**) 4-NPO, used as a QSI reference.

A chemical library of 240 molecules was generated based on AHLs and known QSI structures; it consisted in carboxamides, sulfonamides, sulfonylurea, reverse amides, triazoles, tetrazoles, bromoenamines, bromofuranones and imidazolium derivatives (see experimental section). This library was screened with the above described QS signal-biosensor *P. atrosepticum* CFBP6276-EI (pME6031-*rsmA::uidA*) in the presence of 3-OC8-HSL at 1.5 μM. Using the compounds of the library at 100 μM, 67 putative QSIs were found to restore glucuronidase activity in the *Pectobacterium* QS-biosensor in the presence of AHLs. In the course of this screening, 4-nitropyridine-*N*-oxide (4-NPO) was used as a control QSI (Figure 1) [17]. The identified compounds were thereafter tested at lower concentrations (50, 10, 2.5, and 0.1 μM). At 10, 2.5, and 0.1 μM, none variations of the reporting activity were observed. At 50 μM, the higher glucuronidase activities were measured in the presence of the compounds 29-L-A06, 29-L-A11, 29-L-B02 and 29-L-C03. All of these compounds were imidazolium-derivatives which exhibit bis-*N*-substitution with a polyaromatic group and an aliphatic chain (Figure 1). These compounds were designed by analogy to calmidazolium previously identified as QSI by virtual screening [21]. Their synthesis involved two successive *N*-alkylation of imidazole, with variations in the aromatic moiety (halogenations, fluorenyl) on one nitrogen atom, and variations in the alkyl chain length on the other nitrogen atom. Both substitutions were found to influence the QSI activity when tested in a modified *E. coli* strain which expresses the *Vibrio fisheri* QS-system. Indeed, a stronger QSI-activity was found for shorter chains when the aromatic residue was larger (highly halogenated), or for longer chains when the aromatic residue was smaller (unsubstituted or sterically constrained) [22].

### 2.3. Biological Effects of the Identified QSIs on *Pectobacterium* Cells

For the calculation of the half maximal activity concentration (AC_50_), the activity of the reporter gene *uidA* was measured in the presence of different concentrations of QSIs (0.1 to 100 μM). In addition, cell density (OD_600_) of the cultures was measured in the absence and presence of the QSIs at the AC_50_ concentrations. These values were used to calculate a growth index (GI_AC50_) and evaluate growth inhibition of the QSIs; a ratio value of 1 indicates that the growth of the bacteria is not affected by the presence of the QSI added at the AC_50_ concentration. The AC_50_ values of the four imidazolium-compounds ranged between 14 and 20 μM (Table 1). The GI_AC50_ values (from 0.93 to 0.99) were not statistically different (Kruskal Wallis test α = 5%) from those of the control cultures without QSIs (GI_control_ = 1.00), suggesting that the cell growth was not affected near the AC_50_ concentrations. As a reminder, the AHL concentration in this assay was strictly controlled by the addition of pure 3-OC8-HSL at 1.5 μM in the culture medium, hence the reporting activity of the *Pectobacterium* AHL-biosensor could not be altered by a variation of the AHL level. Moreover, an antibacterial activity should decrease glucuronidase activity by killing the cells; by contrast, imidazolium derivatives increase this reporting activity which is the opposite effect of potential antibacterial activity. All these observations allow us to suggest that the identified molecules could act as QSIs under our experimental conditions. We also observed that the already known QSI 4-NPO that was active in *P. aeruginosa* [23] was less efficient than were the identified imidazolium-derivatives against the QS-regulated gene *rsmA::uidA* of *Pectobacterium*.

The minimal inhibitory concentration (MIC) and minimal bactericidal concentration (MBC) were measured for all QSIs in *P. atrocepticum*. The QSI 29-L-B02 exhibited MIC and MBC values lower than the AC_50_ value, while the other QSIs exhibited MIC and MBC values higher than AC_50_ values, or comparable in the case of the MIC of 29-L-A11 (Table 1). It should be noticed that the MIC and MBC values were measured after 40-h of culture in the presence of QSIs, hence at the end of the growth cycle of the bacteria when nutrients became limiting. In contrast, GI_AC50_ and AC_50_ values were measured during exponential growth of the bacteria. The apparent higher sensitivity of the *Pectobacterium* cells when grown under MIC and MBC conditions as compared to GI_AC50_ and AC_50_ conditions could be explained by the physiological status of the cells.

### 2.4. QSIs Could Moderate the *P. atrosepticum*-Induced Symptoms in Potato Tubers

**Figure 2:**
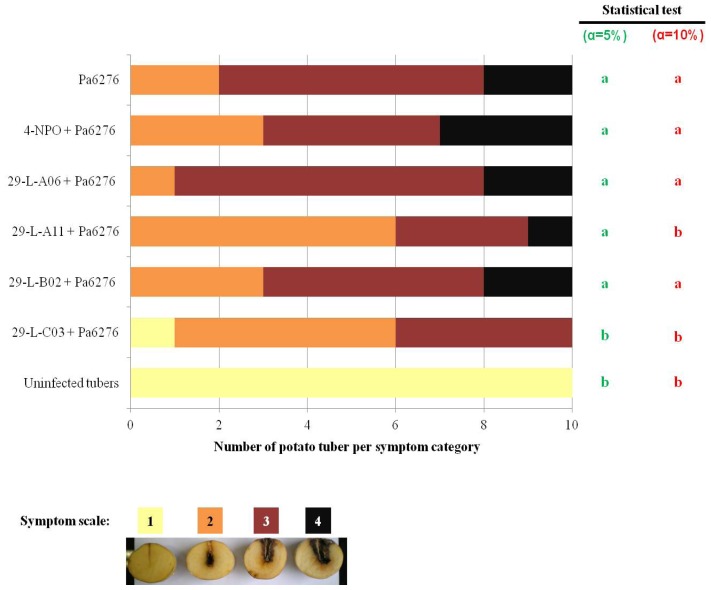
QSIs and soft-rot tuber assay. Each tuber of *S.tuberosum* was inoculated with 10^7^ cells of *P. atrosepticum* CFBP6276 (Pa6276) in the absence and presence of the QSIs at 20 μM. The uninfected tubers were used a negative control. The virulence symptoms are categorized according a four-category scale. The different letters indicate the symptoms of QSI-treated conditions which were statistically different to those obtained in the presence of Pa6276 alone (Kruskal and Wallis test, α = 5% or 10%).

The four QSIs were tested for their capacity to limit the QS-associated symptoms induced by the plant pathogen *P. atrosepticum* CFBP6276 on potato tubers (Figure 2). The QSI 29-L-B02 that exhibited MIC and MBC values lower than AC_50_, did not protect the tubers against the plant pathogen, as the severity of the symptoms was similar to that observed in the absence of QSI (Figure 2). This observation suggested that under the tested conditions the introduced bacterial cells (10^7^ cells at the infection site) were still able to multiply and express the QS-regulated virulence factors in the tuber assay, even in the presence of a potential bacteriostatic and bactericidal delivery of the inhibitory molecule at 20 μM. By contrast, two other QSIs, 29-L-A11 and 29-L-C03 that exhibited a lower bacteriostatic and bactericidal activity than 29-L-B02, reduced (but did not abolish) the severity of the symptoms (Figure 2). The limitation of QS-dependent symptoms was therefore not correlated with the potential bacteriostatic and bactericidal activity of the identified compounds, and could reflect their QSI-activity.

These imidazolium-derivatives were also efficient at the μM range to disrupt QS-signaling in the marine bacterium *Vibrio fisheri* that uses 3-oxo-hexanoyl-L-homoserine lactone as a QS-signal [19]. This feature suggests that they may be used as a structural backbone for the generation of broad range QSIs. Polyaromatic compounds have been frequently described as QSIs. As natural compounds, they have been identified in many organisms, especially plants [14]. As synthetic compounds, they have been revealed by chemical library and virtual (*in silico*) screenings [14,21].

This work extends the spectrum of QSIs targeting the QS-controlled virulence of the plant pathogen *Pectobacterium* [11–13]. Aside the *P. atrosepticum* and *P. carotovorum* species in which QS plays a key-role in virulence, QS has been also involved in a partial regulation of virulence in *D. dianthicola* and the emerging pathogen *Dickeya solani*, which are other causative agents of the soft-rot and blackleg diseases in potato cultures [3]. The QSI-treatment may be proposed as a complement of other QS-targeting approaches such as the use of biocontrol agents and transgenic plants which are able to degrade the QS-signals [7–10]. All the proposed anti-QS strategies remain to be evaluated under green house and field conditions.

## 3. Experimental Section

### 3.1. Bacterial Strains and Growth Conditions

*P. atrosepticum* CFBP6276 and its derivative CFBP6276-EI in which the *expI* gene was disrupted [19] were cultivated in TY medium (tryptone 5 g/L, yeast extract 3 g/L). The *Pectobacterium* QS-biosensor was obtained by electroporating the constructed plasmid pME6031-*rsmA::uidA* in the *expI* mutant CFBP6276-EI. Antibiotics were used at the following concentrations: kanamycin, 50 μg/mL; tetracycline, 10 μg/mL.

### 3.2. Chemical Library

The chemical library of the ICBMS (Université de Lyon, INSA, Villeurbanne, France) contained 240 synthetic derivatives of AHLs or known QSIs. These chemicals were kept in DMSO stock solutions (10 mM) at −20 °C. The library includes various types of QS agonists or antagonists, either structurally related to AHL (carboxamides, sulfonamides, urea, sulfonylurea, reverse amides, triazoles or tetrazoles), or bromoenamines and bromofuranones designed by analogy to natural compounds known as QSI [24–31]. This latter category includes the imidazolium derivatives found to be active in this study and designed as analogues of calmidazolium which were identified as QSIs by virtual (*in silico*) screening [21,22].

### 3.3. Screening for QSIs

Compounds of the chemical library were individually assayed for QSI-activity at two concentrations (100 μM and 0.1 μM) in 96-microwell plates in the presence of the AHL 3-OC8-HSL at 1.5 μM and the *Pectobacterium* QS-biosensor. After 4 h of incubation at 30 °C, β-glucuronidase activities were measured using the appropriate substrate 4-nitrophenyl-β-d-glucuronide, as previously described [32]. The 4-nitropyridine-*N*-oxide (4-NPO) was used as a QSI reference [23]. The added DMSO did not exceed 5% of the total volume of culture medium and did not alter the bacterial growth.

### 3.4. Measurement of AC_50_, GI_AC50_, MIC and MBC Values of the QSIs

In the case of the *Pectobacterium* QS-biosensor, half maximal activity concentrations (AC_50_) were calculated using QSI concentrations ranging from 0.1 to 100 μM. At the AC_50_ concentrations, the growth index (GI_AC50_) was calculated as the ratio of the OD_600nm_ mean-values measured for bacterial cultures performed with and without QSI. Toxicity of these compounds was also evaluated by measuring the minimal inhibitory concentration (MIC) and minimal bactericidal concentration (MBC). MICs, which are the lowest concentrations of QSI inhibiting any visible growth after 40 h of incubation at 30 °C, were estimated by culturing 10^5^ CFU/mL of the *Pectobacterium* cells in the presence of different concentrations of QSI. MBCs, which are the lowest concentrations of QSI (μM) that result in a 99.9% reduction of the initial bacterial population (10^5^ CFU/mL) after 40 h of incubation at 30 °C in the presence of different concentrations of QSI, were estimated by plating 100 μL of the *Pectobacterium* cultures onto agar TY plates. After an incubation of 24 or 48 h at 30 °C, CFU were enumerated and the MBC values calculated.

### 3.5. Virulence Assays on Potato Tubers

Potato tubers of *S. tuberosum* var. Bintje (length 35 to 45 mm, CNPPT/SIPRE, Achicourt, France) were surface sterilized by washing in a diluted commercial bleach solution for 10 min. Next, the potatoes were rinsed once with sterile water and allowed to dry at room temperature overnight. An overnight culture (25 °C; 200 rpm) of the *P. atrosepticum* wild-type strain CFBP6276 in TY medium was collected by centrifugation (room temperature, 4000 rpm, 15 min) and washed twice using 0.8% NaCl. The bacterial pellet was resuspended in 0.8% NaCl (room temperature, 4000 rpm, 15 min). Each tuber (*n* = 10 per conditions) was inoculated with 10^7^ CFU of *P.atrosepticum* in presence of the QSIs at 20 μM. The infected tubers were incubated at 25 °C in a water saturated atmosphere. Five days post-infection, the tubers were cut in the middle, photographed and the soft-rot symptoms were categorized using a virulence scale that contained four categories, depending on the diameter (D) of the maceration zone around the infection site: 1, no maceration; 2, low maceration (D < 2 mm); 3, moderate maceration (D < 5 mm) and 4, strong maceration (D ≥ 5 mm). The Kruskal and Wallis statistical test with α = 5 or 10% allowed the statistical analysis of symptoms on potato tubers.

## 4. Conclusions

Our work highlighted a novel family of QSI that limit *Pectobacterium*-induced symptoms in the potato tubers. The identified QSIs are *N*,*N*′-bisalkylated imidazolium salts which exhibited QSI-activity when used under sub-lethal concentrations. Future works should evaluate the QSI strategy under greenhouse and field conditions, especially in combination with biocontrol-strategies [33–36] to limit the symptoms caused by the pathogens *Pectobacterium* and *Dickeya*.

## Figures and Tables

**Table 1:** Biological characteristics of the four identified QSIs.

	*Pectobacterium* cells			

Name	AC_50_^a^	GIAC_50_^b^	MIC^a^	MBC^a^
**4-NPO**	>100 (12%) c	1.00	50	>100
**29-L-A06**	18	0.94	25	25
**29-L-A11**	14	0.93	10	25
**29-L-B02**	16	0.99	<2.5	2.5
**29-L-C03**	20	0.93	25	25

a Values are expressed in μM;

b Growth index (GI_AC50_) is the ratio of cell densities of bacterial cultures performed in the presence of QSIs at the AC_50_ concentrations to those obtained without QSI;

c In brackets, inhibition (%) at 100 μM, which is the maximal concentration tested in this study.

## Abbreviations

3-OC8-HSL: 3-oxo-octanoyl-l-homoserine lactone
4-NPO: 4-nitropyridine-*N*-oxide
AC_50_: half maximal activity concentration
AHL: *N*-acyl homoserine lactone
DMSO: dimethylsulfoxide
GI_AC50_: growth index at the AC_50_ concentration
MBC: minimal bactericidal concentration
MIC: minimal inhibitory concentration
OD_600_: optical density at a wavelength of 600 nm
Pa6276: *P. atrosepticum* CFBP6276
QS: quorum-sensing
QSI: quorum sensing-inhibitor
